# Plant graph-based pangenomics: techniques, applications, and challenges

**DOI:** 10.1007/s42994-025-00206-7

**Published:** 2025-03-28

**Authors:** Ze-Zhen Du, Jia-Bao He, Wen-Biao Jiao

**Affiliations:** 1 National Key Laboratory for Germplasm Innovation and Utilization of Horticultural Crops, Huazhong Agricultural University, Wuhan, 430070 China; 2 Hubei Hongshan Laboratory, Wuhan, 430070 China; 3 Hubei Key Laboratory of Agricultural Bioinformatics, College of Informatics, Huazhong Agricultural University, Wuhan, 430070 China

**Keywords:** Crop breeding, Genome graph, Genotyping, Pangenome, Structural variation

## Abstract

Innovations in DNA sequencing technologies have greatly boosted population-level genomic studies in plants, facilitating the identification of key genetic variations for investigating population diversity and accelerating the molecular breeding of crops. Conventional methods for genomic analysis typically rely on small variants, such as SNPs and indels, and use single linear reference genomes, which introduces biases and reduces performance in highly divergent genomic regions. By integrating the population level of sequences, pangenomes, particularly graph pangenomes, offer a promising solution to these challenges. To date, numerous algorithms have been developed for constructing pangenome graphs, aligning reads to these graphs, and performing variant genotyping based on these graphs. As demonstrated in various plant pangenomic studies, these advancements allow for the detection of previously hidden variants, especially structural variants, thereby enhancing applications such as genetic mapping of agronomically important genes. However, noteworthy challenges remain to be overcome in applying pangenome graph approaches to plants. Addressing these issues will require the development of more sophisticated algorithms tailored specifically to plants. Such improvements will contribute to the scalability of this approach, facilitating the production of super-pangenomes, in which hundreds or even thousands of de novo–assembled genomes from one species or genus can be integrated. This, in turn, will promote broader pan-omic studies, further advancing our understanding of genetic diversity and driving innovations in crop breeding.

## Introduction

Over the past several decades, the rapid development of advanced DNA sequencing technologies and the dramatic reduction in sequencing costs have revolutionized plant genomic research. This noteworthy progress has allowed the de novo assembly of hundreds of plant genomes and has facilitated extensive-omics studies, including resequencing, transcriptomics, and epigenomics (Jiao and Schneeberger 2017; Xie et al. 2024). These genomic studies often involve mapping sequencing reads against a reference genome. However, due to the existence of sequence diversity throughout populations, the use of a single reference genome may lead to mapping biases or failures, particularly in highly divergent regions or regarding sequences absent from the reference genome. To address these limitations, the concept of the pangenome has emerged as a powerful tool. By incorporating non-reference alleles or sequences from multiple individuals, the pangenome enables accurate mapping of sequencing reads across divergent regions and provides high-resolution insights into sequence diversity.

The term *pangenome* was initially coined to represent the collective gene repertoire from different strains of a bacterial species (Tettelin et al. 2005). Later, it was extended to eukaryotic genomes and defined as the entirety of DNA sequences present in a species. A pangenome typically contains two parts: the core genome (sequences shared by all individuals) and the dispensable genome (sequences present in only a subset of individuals or even a single individual). The first plant pangenome was constructed using seven de novo–assembled soybean genomes that were produced based on short-read sequencing (Li et al. 2014). Over the past decade, driven by the considerable reduction in the cost of sequencing technologies, especially long-read sequencing, more than forty plant pangenome studies have been performed. These efforts have been comprehensively reviewed in the recent literature (Mishra et al. 2024; Schreiber et al. 2024; Shi et al. 2023; Wang et al. 2023).

In principle, a pangenome can be modeled at either the homologous gene level or the DNA sequence level. The former strategy typically involves searching for homologous genes across individual genomes, whereas the latter strategy can be implemented as either a linear reference genome supplemented with the non-reference genome sequences (linear pangenome) or a graph-based pangenome, as described below (Bayer et al. 2020). A linear pangenome can be built by iteratively mapping sequence reads from each individual against a reference genome and assembling unaligned reads. Alternatively, non-reference sequences can be identified using alignments between the reference and de novo assemblies of individual genomes. However, linear pangenomes have limitations both in representing sequence variants and in integrating non-reference sequences into the reference coordinate system (Eizenga et al. 2020). By contrast, pangenomes integrate the reference assembly with all non-reference sequences into a graph, enabling more efficient and accurate read mapping and variant calling (Paten et al. 2017). To date, the graph pangenome has been widely applied to many plant species, including the model species *Arabidopsis thaliana* (Kang et al. 2023), and major crops such as rice (Qin et al. 2021; Shang et al. 2022), tomato (Li et al. 2023; Zhou et al. 2022), and soybean (Liu et al. 2020; Zhang et al. 2024a). In addition to identifying previously inaccessible genetic variants, graph pangenome studies also facilitate crop breeding by enabling comprehensive variant-based genetic mapping and appropriate genome selection (He et al. 2023; Liu et al. 2024a; Zhou et al. 2022).

In practice, these studies normally follow a workflow that includes pangenome graph construction, sequencing read mapping, variant genotyping, and genome-wide association study (GWAS) or genome selection. However, the efficient construction and application of graph pangenomes in plants face several challenges. These challenges arise from the intrinsic complexities of plant genomes, including large genome sizes, excessive amounts of repetitive sequences and transposable elements, high heterozygosity, and polyploidy. In this review, we focus on recent advances in creating useful algorithms for constructing pangenome graphs and downstream analyses for variant calling and genotyping, along with their application in plant pangenomic studies. In addition, we discuss the challenges associated with pangenome graph-based variant genotyping in plants and propose potential solutions to address these challenges.

## Construction of a pangenome graph

**Fig. 1:**
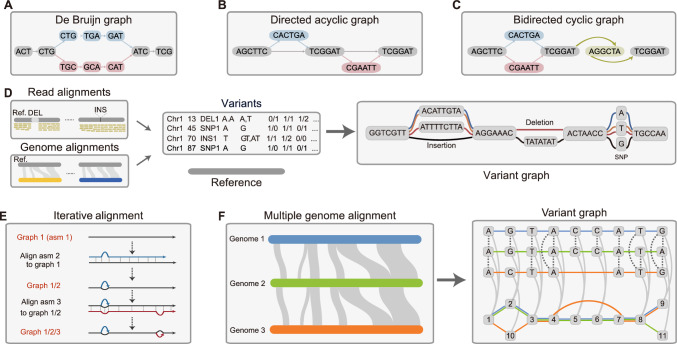
Pangenome models and construction of a pangenome graph. **A–C** Typically, pangenome graphs can be represented as de Bruijn graphs (**A**), sequence graphs, or variant graphs. Sequence graphs and variant graphs can be structured as directed acyclic graphs (DAGs) (**B**) or bidirected cyclic graphs (BCGs) (**C**). **D** Most pangenome graph tools construct a variant graph based on a reference genome and sequence variants derived from read alignments or assembly comparisons. **E** Construction of a pangenome graph using Minigraph involves an iterative process of genome alignments. asm, assembly. **F** Construction of a pangenome graph using tools such as seqwish or the PGGB pipeline directly based on multiple genome alignments

The construction of a pangenome graph is a fundamental, yet often time-consuming, step for pangenomic studies. Currently, there is no fully satisfactory method to represent and construct pangenome graphs. Algorithm developers must consider several key issues: the efficiency of graph construction models in terms of run time and memory usage, cost, scalability and stability when dealing with hundreds or even thousands of genomes, the design of fast indexing methods for useful and informative graph manipulations, and efficiency in memory usage for storage of the graph. They must also consider the method used for read-to-graph alignment, creation of an output format that is both easily readable by biologists and suitable for genome-wide or local visualization, and compatibility with other downstream bioinformatic analyses or the traditional linear reference system (Andreace et al. 2023; Eizenga et al. 2020; Minkin et al. 2017). One specific challenge is whether and/or how to embed the original linear coordinates and sample information of sequences into the graph. Technically, there are two widely used computational structures for representing pangenome graphs: the de Bruijn graph and the sequence graph (Fig. 1).

### de Bruijn graphs

The de Bruijn graph (DBG) is commonly utilized in genome assemblers; the nodes represent overlapping k-mers generated from billions of short reads (Fig. 1A). Typically, the size of k-mers is fixed, and connected nodes share an overlap of *k* − 1 bases. In the context of pangenome graphs, these graphs can be built from multiple assembled genomes using modified data structures. These include colored DBGs [e.g., Cortex (Iqbal et al. 2012) and Bifrost (Holley and Melsted. 2020)], succinct DBGs [e.g., (Baier et al. 2016)], compacted DBGs [e.g., TwoPaCo (Minkin et al. 2017), BCALM2 (Chikhi et al. 2016), and Bifrost], bloom filter based DBGs (Salikhov et al. 2014), Minimizer-space DBGs [e.g., mdbg (Ekim et al. 2021)] or the integration of one or more of these [e.g., Bifrost, SplitMEM (Marcus et al. 2014), and PanTools (Sheikhizadeh et al. 2016)]. The main limitations of these types of representation are the use of a fixed k-mer length, complex graph topologies, and incompatibility with the variation graph model due to node overlaps (Eizenga et al. 2020).

### Sequence graphs

Currently, the sequence graph is more extensively used because a number of downstream bioinformatic tools are available and are compatible with traditional linear reference-based analyses such as variant calling and genotyping. In a sequence graph, nodes are labelled with DNA sequences, and edges denote the successive relationships of sequences in the genome. A path in this graph corresponds to a haplotype sequence of incorporated genomes, and bubbles can signify variations among genomes (Fig. 1B, C). When nodes represent both strands of DNA, the graph is considered to be bidirected. One type of sequence graph, a variation graph, embeds a set of paths representing sequences of a pangenome (Garrison et al. 2018).

### Reference variant-based sequence graphs

There are two main approaches for constructing a sequence graph (Table 1). The first approach, denoted as reference variant-based, typically constructs a pangenome graph using a directed acyclic graph (DAG) or a bidirected cyclic graph (BCG) based on a linear reference genome and a collection of different types of variants from a given population (Fig. 1D). Tools implementing this approach include Graphtyper (Eggertsson et al. 2019), BayesTyper (Sibbesen et al. 2018), Paragraph (Chen et al. 2019), Seven Bridges’ Graph Genome Pipeline (Rakocevic et al. 2019), and Gramtools (Letcher et al. 2021). Most of these tools involve constructing a pangenome graph using inputs from a linear reference genome in a FASTA format file and variants in a VCF format file. Additionally, vg and HISAT2 (Kim et al. 2019) support the same input files but opt for a bidirected cyclic variation graph, which may require more computational resources. However, the VCF format can sometimes fail to fully represent complex structural variations, such as long insertions nested with small variants (Danecek et al. 2011; Li et al. 2020). Moreover, the quality of the input variants can greatly impact the efficiency of the pangenome graphs. Input variants are often obtained via assembly-comparison and long-read mapping-based methods, or a combination of these methods. However, the quality of these variants can be affected by residual alignment errors, false variant calling, or variant redundancy. For instance, some structural variants (SVs) in the input variant files may have only tiny differences in their breakpoints due to inaccuracies in SV calling (Audano and Beck. 2024; Cameron et al. 2019; Liu et al. 2024b).

**Table 1:** Tools for pangenome graph construction

Tools	Graph types	Input data	Indexing method	Graph format	References
Minigraph	DAG	Multi-ASM	Minimizer	rGFA	Li et al. (2020)
Seqwish/PGGB	DAG	Multi-ASM	CSA	GFA	Garrison et al. (2023)
Minigraph-Cactus	DAG	Multi-ASM	Minimizer	GFA, VCF	Hickey et al. (2024)
Graphtyper	DAG	Ref. + Var.	k-mer hash table	GFA	Eggertsson et al. (2019)
vg	BCG	Ref. + Var.	GCSA, GBWT	vg, GFA, GBZ	Garrison et al. (2018)
BayesTyper	DAG	Ref. + Var.	k-mer hash table	GFA	Sibbesen et al. (2018)
Paragraph	DAG	Ref. + Var.	k-mer hash table	GFA	Chen et al. (2019)
Gramtools	DAG	Ref. + Var.	vBWT	GFA	Letcher et al. (2021)

*BCG* bidirected cyclic graph, *DAG* directed acyclic graph, *Ref. + Var.* reference genome sequences plus genetic variants, *Multi-ASM* multiple genome assemblies, *CSA* compressed suffix array, *GCSA2* generalized compressed suffix array, *GBWT* graph Burrows-Wheeler transform, *Vg map* uses GCSA2, whereas vg giraffe uses GBWT, *vBWT*, variation-aware Burrows–Wheeler transform, *GBZ* a file format for pangenome graphs based on the data structures used in the vg giraffe aligner and GBWT index, *GFA* graphical fragment assembly, *rGFA* reference GFA, *VCF* variant call format; vg, file format of pangenome graphs with the vg suffix generated using vg

### Genome alignment-based sequence graphs

A second method, referred to as multiple genome alignment, uses multiple iterative genome sequence alignments to produce a pangenome graph (Fig. 1E, F). Multiple sequence alignments generated by tools such as Cactus/ProgressiveCactus (Armstrong et al. 2020), and SibeliaZ (Minkin and Medvedev 2020) can reflect sequence relationships and can be further rendered into pangenome graphs. However, whole-genome multiple alignment remains a considerable computational challenge, especially as the number of sequenced genomes increases. To circumvent the need for direct multiple alignments, Minigraph (Li et al. 2020) extends the minimap2 linear-reference alignment algorithm to perform rapid sequence-to-graph alignments using an iterative procedure to construct a graph by mapping each genome assembly to an existing graph under a pangenome graph model known as reference Graphical Fragment Assembly (rGFA) (Fig. 1E, Table 1). Minigraph can rapidly construct a pangenome graph for 20 human genomes in 3 h with a peak memory usage of 98 GB (Li et al. 2020). However, it constructs graphs that include only large variants (default value > 50 bp), it cannot align sequences to a graph containing all small variants, and it does not encode sample information. Recently, the Minigraph-Cactus pipeline (Hickey et al. 2024) combines the advantages of Minigraph and Cactus and an improved version of vg to generate base-level pangenome graphs that incorporate all types of genetic variations directly from whole-genome alignments. This pipeline can be used to generate pangenome graphs including 90 human haplotypes, and they can complete the construction and indexing in 3 days. Additionally, this pipeline supports and enhances the performance of multiple downstream analyses, including read mapping and variant calling.

Unfortunately, pangenome graphs created by Cactus, Minigraph, or Minigraph-Cactus are not entirely unbiased, as they depend on an initial reference genome or a phylogenetic guide tree, which can influence the construction of a pangenome graph (Garrison and Guarracino 2023). Alternatively, algorithms such as seqwish (Garrison and Guarracino 2023) build a pangenome variant graph directly from the given genome sequences and their alignments (Fig. 1F). Despite high computational costs, seqwish can uniformly represent sequence relationships, can embed all variants in the resulting graph, and can support the reconstruction of all input sequences. It is also wrapped into a graph pangenome pipeline known as PanGenome Graph Builder (PGGB) (Garrison et al. 2023). This pipeline uses wfmash (Guarracino et al. 2021) for all-vs-all alignment, creates a graph with seqwish, iterates (three times by default) the graph using smoothxg, and removes node redundancy with gfaffix. Another reference-free multiple genome aligner, ACMGA, which is specialized for plant species, shows promise for integration into the graph pangenome workflow (Zhou et al. 2024). Notably, PGGB, Minigraph, and Minigraph-Cactus have all been tested during building of a draft human pangenome graph by the Human Pangenome Reference Consortium (HPRC) that is based on 47 phased and diploid assemblies (Liao et al. 2023). As expected, the PGGB pipeline produces the largest pangenome graphs in terms of both the number of nodes and edges and the total length of embedded sequences. A recent study compares five pangenome graph construction methods (Andreace et al. 2023), including two DBG-based methods: Bifrost and mdbg, and three genome alignment-based methods: Minigraph, Minigraph-Cactus, and PGGB. Further innovations in plant genome analysis and algorithm construction will provide insights into their performance and help users select the appropriate approach for plant studies. With the advent of long-read sequencing technologies, which enable efficient acquisition of high-quality (even telomere-to-telomere) assemblies, genome alignment-based methods present a promising avenue to accelerate future large-scale graph pangenome studies involving hundreds of de novo assemblies.

## Pangenome graph-based read alignment and variant genotyping

Because pangenome graphs integrate multiple individual genome sequences, they can improve the performance of downstream genomic analyses, including variant calling and genotyping. A typical workflow of such analyses generally involves three main steps: graph construction and indexing, read alignment or k-mer searching, and variant genotyping. In many pangenomic studies, high-quality pangenome graphs and SV collections are often generated based on long-read assemblies of multiple representative individual genomes. The number of individual genomes genotyped often reaches the hundreds or thousands, and these individual genomes are frequently sequenced using short reads. After constructing and indexing a pangenome graph, reads from each individual are aligned to the graph with read-to-graph aligners (mapping-based). Alternatively, a k-mer-based approach can be used, in which specific k-mers of each variant in the pangenome graph are searched for in the reads. Finally, variant genotypes are typically determined using probabilistic models that fit the count of reads or k-mers supporting the reference and variant alleles.

Currently, there are more than ten pangenome graph-based genotypers, including mapping-based tools (Table 2) such as vg (Garrison et al. 2018), Paragraph (Chen et al. 2019), Gramtools (Letcher et al. 2021), HISAT-genotyper (Kim et al. 2019), and GraphTyper (Eggertsson et al. 2019), as well as k-mer-based tools (Table 3) such as BayesTyper (Sibbesen et al. 2018) and PanGenie (Ebler et al. 2022). There is also an ensemble genotyping tool, EVG, which combines both approaches. Notably, most graph-based variant tools support only genotyping, and few tools can identify novel variants based on read alignments against pangenome graphs (Table 3). Tools such as vg can detect a wide spectrum of novel variants using short reads, whereas SVarp (Söylev et al. 2024) specializes in detecting structural variants using long sequencing reads.

**Table 2:** Tools for read-to-graph alignment

Tools	Reads	Input graph	Graph format	Output format	References
GenomeMapper	Short	DAG	NA	BED, SHORE	Schneeberger et al. (2009)
deBGA	Short	DBG	NA	SAM	Liu et al. (2016)
BGREAT	Short	DBG	NA	TSV	Limasset et al. (2016)
BrownieAligner	Short	DBG	NA	TSV	Heydari et al. (2018)
Seven Bridges	Short	DAG	NA	BAM	Rakocevic et al. (2019)
Paragraph	Short	DAG	GFA	JSON	Chen et al. (2019)
HISAT2	Short	BCG	NA	SAM	Kim et al. (2019)
vg giraffe	Short	BCG	vg, GFA, GBZ	GAM, BAM	Sirén et al. (2021)
vg map	Short, long	BCG	vg, GFA, GBZ	GAM, BAM	Hickey et al. (2020)
PaSGAL	Short, long	DAG	vg, txt	TSV	Jain et al. (2019)
AStarix	Short, long	DAG	GFA	TXT	Ivanov et al. (2022)
GraphAligner	Long	DBG, BG	vg, GFA	GAM, JSON, GAF	Rautiainen and Marschall (2020)
SPAligner	Long	DBG	GFA	TSV, FASTA, GPA	Dvorkina et al. (2020)
Minigraph	Long	BG	GFA	GAF	Li et al. (2020)
Minichain	Long	DAG	GFA	PAF, GAF	Chandra et al. (2024)
GraphChainer	Long	DAG	vg, GFA	GAM, JSON	Ma et al. (2023)
PanAligner	Long	DAG	GFA, rGFA	GAF	Rajput et al. (2024)

*BCG* bidirected cyclic graph, *BG* bidirected graph, *DAG* directed acyclic graph, *DBG* de Bruijn graph

**Table 3:** Tools for pangenome graph-based genotyping and novel variant calling

Tools	Methods	Calling	Genotyping	References
Paragraph	Read mapping	–	SNP, indel, SV	Chen et al. (2019)
Seven Bridges	Read mapping	–	SNP, indel, SV	Rakocevic et al. (2019)
HISAT-genotype	Read mapping	–	SNP, indel	Kim et al. (2019)
GraphTyper2	Read mapping	SNP, indel	SNP, indel, SV	Eggertsson et al. (2019)
vg	Read mapping	SNP, indel, SV	SNP, indel, SV	Hickey et al. (2020)
Gramtools	Read mapping	–	SNP, indel, SV	Letcher et al. (2021)
Minos	Read mapping	–	SNP, indel	Hunt et al. (2022)
BayesTyper	k-mers comparisons	–	SNP, indel, SV	Sibbesen et al. (2018)
PanGenie	k-mers comparisons	–	SNP, indel, SV	Ebler et al. (2022)
KAGE	k-mers comparisons	–	SNP, indel	Grytten et al. (2022)
EVG	k-mers and mapping	–	SNP, indel, SV	Du et al. (2024)

EVG integrates results from both k-mer comparison-based tools and read mapping-based tools

### Mapping-based genotyping

Mapping-based genotyping methods typically determine genotypes based on the alignment of reads to pangenome graphs. The aligners allow aligning reads to a de Bruijn graph [e.g., deBGA (Liu et al. 2016), BGREAT (Limasset 2016), BrownieAligner (Heydari et al. 2018)] or a sequence graph structured as a DAG [e.g., GenomeMapper (Schneeberger et al. 2009), Seven Bridges (Rakocevic et al. 2019), Paragraph (Chen et al. 2019)] or as arbitrary variant graphs [e.g. vg map (Garrison et al. 2018), vg giraffe (Sirén et al. 2021), GraphAligner (Rautiainen and Marschall 2020)]. Some of these tools are designed to handle both short and long reads, including vg map, PaSGAL (Jain et al. 2019), and AStarix (Ivanov et al. 2022). Others, such as GraphAligner (Rautiainen and Marschall 2020) and PanAligner (Rajput et al. 2024), are tailored for long-read alignments (Table 2). Their alignment outputs can be further used for genotyping using tools such as vg.

Most read-to-graph aligners (Table 2) implement a seed-and-extend strategy. A significant challenge in these methods is the high computational cost involved in aligning all reads to a graph. For example, the mapping speed of the old version of vg (vg map) is an order of magnitude slower than state-of-the-art linear reference-based aligners. This can lead to excessive use of computational resources when pangenome graphs become highly complex due to a large number of genomes and variants. A faster version of vg (vg giraffe) significantly reduces the search space of possible haplotypes by prioritizing paths observed in individual genomes. This optimization enables vg giraffe to achieve much faster performance, making it comparable in speed to single linear reference-based aligners while maintaining high accuracy (Sirén et al. 2020, 2021). Other tools, such as GraphTyper2, speed up the mapping process and reduce the computational cost by remapping pre-aligned reads (those aligned against a linear reference genome) to local graphs of genome bins (default size of 1 Mb) (Eggertsson et al. 2019). Similarly, the Paragraph also utilizes the pre-mapping result of reads to rapidly locate the candidate regions and remap reads to the graph (Chen et al. 2019).

### k-mer comparison-based genotypers

Unlike mapping-based methods, k-mer-based genotyping tools (Table 3) bypass the need for aligning reads to a graph, thereby outperforming mapping-based approaches in terms of speed and use of computational resources. Normally, they count the frequency of variant locus-specific k-mers by scanning the sequencing reads, and they then model the distribution of these frequencies while accounting for sequencing errors. This allows them to infer genotypes by calculating or maximizing the likelihood of each candidate haplotype (a combination of several adjacent variants). These genotypers differ in their choice of k-mer size, haplotype length, and statistical models. For example, BayesTyper uses a Poisson distribution to determine the probability for noise k-mers counts and a negative binomial distribution for diplotype (haplotype pair) counts to model the posterior distribution over possible haplotypes (Sibbesen et al. 2018). However, methods that rely solely on unique k-mers often struggle in repetitive regions that lack sufficient unique k-mers. In contrast, another k-mer-based graph genotyper, PanGenie, can improve genotyping performance for large insertions and variants in duplicated regions by leveraging long-range connectivity information from multiple haplotype-resolved assemblies (Ebler et al. 2022). Our recent study has shown its superiority in various plant genomes and sequencing conditions (Du et al. 2024). Notably, combining these tools can further enhance genotyping performance, as demonstrated by EVG (Du et al. 2024).

## Applying graph pangenomes in plant genomics

For many plant crops, substantial numbers of individual genomes have been sequenced using short-read sequencing platforms in conventional population resequencing or pangenome studies. However, the investigation of the genetic diversity of these crops has primarily focused on small variants. In recent years, long-read sequencing technologies have enabled the discovery of previously hidden SVs through de novo assembly or resequencing of multiple representative individuals. With the advent of pangenome graphs, we can now comprehensively reanalyze these short-read sequencing datasets to identify population diversity that encompasses all types of variants. Many graph pangenome studies have identified numerous SVs in large-scale populations and have exploited these SV genotypes, often in conjunction with SNP and indel genotyping, for expression Quantitative Trait Loci (eQTL), GWAS, Marker-Assisted Selection (MAS), and Genome Prediction (GP) (Table 4).

**Table 4:** Summary of plant graph pangenome studies

Species/genus^a^	Genome size (Mbp)	Repeat percentage	Genome assemblies^b^	+ SNPs^c^	SV count	Graph model^d^	Construction	Genotyping	Application^e^	References
*Glycine max*	1011	53%	29 (15C, 10 L, 4W)	No	124,222	Ref. + Var.	vg	vg	GWAS	Liu et al. (2020)
*Brassica rapa*	353	47%	18 (18 C)	No	87,032	Ref. + Var.	vg	vg	GD	Cai et al. (2021)
*Oryza sativa*	373	39%	33 (33 C)	No	164,009	Ref. + Var.	vg	vg	GWAS	Qin et al. (2021)
*Raphanus sativus*	460	47%	11 (8 C, 1 SW, 2 W)	No	53,800	Ref. + Var.	vg	Not done	GD	Zhang et al. (2021)
*Sorghum bicolor*	704	68%	16 (11C, 5 W)	No	unknown	MGA	Minigraph	Not done	GWAS	Tao et al. (2021)
*Cucumis sativus*	244	24%	12 (9 C,3 W)	No	54,107	Ref. + Var.	vg	vg	GWAS	Li et al. (2022)
*Oryza sativa*	373	39%	252 (214 C, 38 W)	No	159,491	MGA, Ref. + Var.	Minigraph, vg	vg	eQTL, GWAS	Shang et al. (2022)
*Solanum lycopersicum*	802	61%	132 (92 C, 40 W)	Yes	195,957	Ref. + Var.	vg	vg	eQTL, GWAS, MAS, GP	Zhou et al. (2022)
*Triticum aestivum*	14,500	85%	16 (16 C)	No	unknown	MGA	Minigraph	Not done	GD	Bayer et al. (2022)
*Zea mays*	2106	85%	722 (508 C, 31 L, 183 W)	Yes	201,502	Ref. + Var.	vg	Not done	eQTL	Gui et al. (2022)
*Arabidopsis thaliana*	134	23%	32 (32 W)	No	61,322	MGA	Minigraph	vg	GWAS	Kang et al. (2023)
*Camellia sinensis*	3060	78%	22 (19 C, 3 L)	No	887,986	Ref. + Var.	vg	vg	GWAS, MAS	Chen et al. (2023)
*Capsicum annuum*	3024	78%	3 (3 C)	Yes	481,604	Ref. + Var.	vg	vg	GWAS	Liu et al. (2023)
*Citrus*	337	41%	16 (3 C, 13W)	No	399,338	Ref. + Var.	vg	vg	GD	Huang et al. (2023)
*Gossypium hirsutum*	2295	64%	12 (9 C, 3 W)	No	182,593	Ref. + Var.	vg	vg	GWAS	Jin et al. (2023)
*Pennisetum glaucum*	1580	77%	11 (4 C, 1 L, 3 W, 3 N)	No	424,085	Ref. + Var.	vg	vg	GWAS	Yan et al. (2023)
*Setaria italica*	401	40%	113 (36 C, 41 L, 36 W)	No	184,429	Ref. + Var.	vg	vg	GWAS, GP	He et al. (2023)
*Solanum lycopersicum*	802	71%	112 (71 C, 41 W)	No	360,189	Ref. + Var.	vg	vg	GWAS	Li et al. (2023)
*Vitis vinifera*	486	49%	9 (9 W)	Yes	unknown	MGA	PGGB	vg	GWAS	Cochetel et al. (2023)
*Brassica napus*	1008	56%	16 (16 C)	No	334,461	Ref. + Var.	Paragraph	Paragraph	eQTL, GWAS	Zhang et al. (2024c)
*Brassica oleracea*	581	61%	27 (25C, 2W)	No	452,148	Ref. + Var.	vg	vg	eQTL, GWAS	Li et al. (2024a)
*Brassica oleracea*	634	59%	11 (10 C, 1 W)	No	469,217	Ref. + Var.	vg	vg	GD	Guo et al. (2024)
*Cicer arietinum*	532	49%	10 (2 C, 8 W)	No	465,535	Ref. + Var.	vg	vg	GD	Khan et al. (2024)
*Citrullus*	369	57%	28 (8 C, 6 L, 14 W)	No	461,987	MGA	Minigraph, vg	BayesTyper	GWAS	Zhang et al. (2024b)
*Glycine max*	1010	51%	30 (16 C, 10 L, 4 W)	No	47,058	Ref. + Var.	vg	vg	GWAS	Zhang et al. (2024a)
*Gossypium arboreum*	1688	79%	15 (15 C)	Yes	22,540	MGA	Minigraph-Cactus, vg	vg	eQTL, GWAS	Li et al. (2024b)
*Gossypium hirsutum*	2318	68%	35 (15 C, 20 SW)	Yes	94,497	MGA	Minigraph-Cactus, vg	vg	eQTL, GWAS	Li et al. (2024b)
*Hordeum vulgare*	4200	88%	77 (18 C, 36 L, 23 W)	No	unknown	MGA	Minigraph, vg	vg	GD	Jayakodi et al. (2024)
*Vitis vinifera*	490	39%	18 (13 C, 5W)	Yes	236,449	MGA	Minigraph-Cactus, PGGB	PanGenie	GWAS, GP	Liu et al. (2024a)

^a^The species or genus name of most genomes in the pangenome graph

^b^Number of assembled genomes used to construct the graph pangenome. C, cultivar; L, landrace; SW, semi-wild; W, wild

^c^Whether small variants (SNPs and indels) are incorporated into the pangenome graph

^d^Model used to represent pangenome graphs, including multiple genome alignment (MGA) and reference together with variants (Ref. + Var.)

^e^Downstream applications of pangenome graphs, including expression quantitative trait loci (eQTL); genetic diversity and/or population genetics (GD); genomic prediction (GP); genome-wide association study (GWAS); and marker-assisted selection (MAS)

### Discovering hidden genetic diversity

In one pioneering study, a graph-based soybean pangenome was constructed using the vg tool, combining de novo assemblies of 29 representative individual genomes (Liu et al. 2020). This pangenome graph contained approximately 124,000 non-redundant SVs and was subsequently used to identify and genotype SVs in a larger population of nearly 2900 accessions with short sequencing reads. Later, a rice pangenome study generated a pangenome graph with 66,542 presence-absence variations (PAVs) selected from 171,012 non-redundant SVs based on 32 *O. sativa* cultivars and one *O. glaberrima* cultivar (Qin et al. 2021). Another more extensive rice graph pangenome was constructed from 252 rice genomes, including cultivars from *O. sativa*, *O. glaberrima*, and wild samples from their progenitors, *O. rufipogon* and *O. barthii* (Shang et al. 2022). This pangenome graph contained 159,491 high-quality deletions and insertions. In addition, this study used the Minigraph tool to create a multi-assembly pangenome graph containing 1.15 Gb of sequences (nearly three times the genome size) that were absent in the reference genome. Unlike these examples, Zhou et al. assembled 31 tomato genomes using PacBio HiFi reads and constructed a preliminary pangenome graph based on all types of variants (SNPs, indels, and SVs) that were identified from these genomes, as well as SVs identified from Oxford Nanopore Technology (ONT) reads from 100 tomato genomes (Zhou et al. 2022). This pangenome graph was expanded to include more than 19 million variants by incorporating additional SNPs and indels identified from short reads of 706 tomato accessions. The final pangenome graph successfully genotyped 8 million variants across 332 tomato accessions. Additionally, in recent years, about 30 graph pangenomes have been released for other cereals (maize, wheat, sorghum, foxtail millet, etc.) and horticultural crops (radish, *Brassica rapa*, tomato, cucumber, pepper, grape, watermelon, etc.) (Table 4).

### Crop domestication and breeding

Beyond capturing a more comprehensive picture of genetic diversity, graph pangenomes help pinpoint the functional loci correlating with crop domestication and agronomic traits. For instance, an early soybean graph pangenome study found that a 10 kb PAV at an *HPS*-encoding gene might control seed luster variation in soybeans (Liu et al. 2020). Significantly, SV-based GWAS can identify candidate functional SVs by integrating genotyping information derived from graph pangenomes with phenotypic data whereas conventional SNP-based GWAS might miss these associations. An example of this was observed in an early rice graph pangenome study that conducted an SV-based GWAS for the leaf senescence phenotype. The authors identified a strongly associated SV, a 987-bp LTR insertion at the upstream of gene *Os06g13470*, that was not detected by SNP-only GWAS (Qin et al. 2021). Other studies have also highlighted the role of pangenome graph-derived SVs or PAVs in crop domestication. A recent pangenome study on foxtail millet not only revealed the importance of PAVs in selection analysis but also provided examples of PAV genes linked to two key domestication traits of cereal crops, seed non-shattering and grain yield (He et al. 2023). In addition, given that a high proportion of SVs are located within promoters or gene sequences, some graph pangenome studies have leveraged SVs and gene expression data to map candidate genes responsible for important agronomic traits (Li et al. 2024a; Liu et al. 2020; Zhou et al. 2022).

Furthermore, pangenome graphs enhance our understanding of missing heritability and empower genomic selection. By analyzing approximately 20,000 molecular traits using graph pangenome-based association studies, Zhou et al. elucidated the contribution of variants, particularly SVs, to heritability, marker-assisted selection, and genomic selection (Zhou et al. 2022). Specifically, the combination of SVs with SNPs and indels facilitated the capture of a greater proportion of missing heritability. Several case studies highlighted the significance of SVs in marker-assisted selection and genomic prediction (Chen et al. 2023; He et al. 2023; Liu et al. 2024a; Zhou et al. 2022). In contrast, traditional linear reference-based analyses often fail to detect or accurately quantify this missing heritability. A graph pangenome study of *Setaria* also underscored the pivotal role of graph pangenomes in genomic selection (He et al. 2023). In this study, the inclusion of both SVs and SNPs led to improvements in predictive accuracy from 0.04% to 12.67% for over 70% of the traits examined compared to SNP-only markers.

### Technical considerations

Most of these studies create pangenome graphs by fusing reference sequences and variations, as vg does. From a technical standpoint, the long-read-based assembly alignment approach can typically yield substantial high-quality variants, especially SVs (Goel et al. 2019). Additionally, calling small variants can also benefit this approach, particularly in duplicated regions (Jaegle et al. 2023). Pangenomic studies in tea and tomatoes have shown that the quality of SVs can be further enhanced when long-read alignment-based methods are included (Chen et al. 2023; Li et al. 2023; Zhou et al. 2022). Moreover, long-read assemblies from a sufficient number of representative individuals are often necessary. Earlier plant graph pangenome studies typically selected fewer than 50 individuals, whereas recent pangenome studies of crops such as tomato, rice, and foxtail millet have collected de novo assemblies from more than 100 genomes. For example, in a graph-based pangenome study on *Setaria*, 110 genomes from wild, landrace, and modern cultivars were assembled using PacBio long-read sequencing, achieving a contig N50 ranging from 127 kb to 5.5 Mb and high levels of assembly completeness (He et al. 2023). These numerous high-quality de novo assemblies facilitate the detection of SVs, pangenome graph construction, and SV-based GWAS and genomic prediction. However, integrating all types of variants from a large population might be computationally intractable for current pangenome graph tools. In practice, incorporating SVs into a pangenome graph alone is apparently acceptable for many pangenome studies, though small variations around SV breakpoints can affect short-read mapping and SV genotyping. Notably, assembly quality in terms of contiguity, completeness, and accuracy is critical for identifying SVs of all sizes as accurately as possible. In addition, telomere-to-telomere assemblies can allow the detection of more high-quality SVs in complex regions such as centromeres and pericentromeres. A recently released watermelon pangenome contained 461,987 nonredundant SVs from 27 telomere-to-telomere assemblies (Zhang et al. 2024b). However, due to the limitations of current algorithms, these complex regions are often ignored or masked in the pangenome graphs (Liao et al. 2023).

### Super-pangenome graphs

Recently, the graph-based pangenome has been extended to the genus, section, or phylogeny-clade level by constructing a super-pangenome graph based on the genomes of cultivar species, wild relatives, and phylogenetically close species. Because wild or closely related species often exhibit higher genetic diversity and better adaptation to biotic and abiotic stress, their untapped genetic diversity offers unprecedented opportunities for crop improvement (Bohra et al. 2022; Khan et al. 2020). As of now, graph super-pangenome studies have been conducted in rice (Shang et al. 2022), maize (Gui et al. 2022), tomato (Li et al. 2023), chickpea (Khan et al. 2024) and *Citrus* (Huang et al. 2023). In a tomato super-pangenome study, researchers constructed a section-wide, graph-based super-pangenome based on 11 assemblies of tomato wild relatives and cultivars, as well as 100 previously reported tomato genomes (Li et al. 2023). By incorporating more genetic variants from wild relatives, they uncovered a substantial number of candidate loci associated with tomato traits and discovered one wild tomato gene that potentially increases yields in cultivars. Another pangenome study in the orange subfamily identified 42,000–81,000 SVs among 12 *Citrus* and related species while building a pangenome graph using the vg tool (Huang et al. 2023). Although functional loci were mapped through SV-based GWAS in these super-pangenome studies, the efficiency of graph pangenome tools in handling such genetically divergent populations remains uncertain. Specifically, short sequencing reads from a cultivar genome may be not aligned well to certain pangenome graph nodes where SVs have been identified from distantly related species. This can lead to failures in SV genotyping and adversely affect downstream applications.

## Challenges in applying graph-based pangenomes to plant genomics

Most current pangenome graph tools have been developed based on human genome datasets. However, significant challenges arise when they are applied to plant genomes due to the wide range of genomic complexities in plants, including repetitive sequence content, large genome sizes, heterozygosity, and ploidy levels. Although there have been multiple graph-pangenome studies in plants, the performance of these graph-based bioinformatic tools should be comprehensively evaluated in different scenarios. A recent benchmark study highlights several challenges in applying graph-based genotyping algorithms to plant genomes (Du et al. 2024).

### Repetitive regions

As many SVs are related to the movements of transposable elements, accurate genotyping in repeat-enriched genomes or locally repetitive regions, such as centromeres and R gene clusters, poses a significant challenge. Specifically, identical or highly similar repeats can introduce multiple candidate alignment positions or erase unique k-mers in a pangenome graph. Consequently, both read alignment-based and k-mer-based genotyping tools struggle to distinguish between possible genotypes. Du et al. have shown that the accuracy and recall of most pangenome graph-based genotypers significantly decrease in repetitive regions, especially for repeat-riche species like maize (Du et al. 2024). Some graph pangenome studies exclude these highly complex regions (Li et al. 2024b; Liu et al. 2024a). For example, the Minigraph-Cactus pipeline marked out centromeric regions when constructing the draft human pangenome reference (Liao et al. 2023). The use of multiple telomere-to-telomere assemblies for graph construction and long, high-fidelity PacBio reads for genotyping could benefit downstream analyses in these challenging regions. For example, with full sequence information, a pangenome graph can help distinguish pseudo-heterozygous variants that arise from inaccurate mapping of reads in highly similar regions (Jaegle et al. 2023). In addition, the development of more sophisticated tools that consider longer k-mers or both unique and repetitive k-mers may help alleviate mapping ambiguities.

### Large genomes and graphs

Most large-scale plant graph pangenome studies focus on species having relatively small genomes or those genomes with a lower percentage of transposable elements. Larger plant genomes, such as wheat, can greatly increase the size of the pangenome graph, slow down read alignments, and require more computational resources. To accelerate the construction of pangenome graphs and the genotyping of variants for these large genomes, some studies incorporate only certain types of variants, such as SVs. However, this approach may diminish the mapping accuracy of reads and impede the correct genotyping of large variants, especially those often accompanied by nearby small variants. Similarly, increasing the number of graphed genomes can impact the running efficiency and genotyping accuracy of graph-based genotypers (Du et al. 2024). More divergent genomes create more bubbles and paths in the pangenome graph, expanding the search space and leading to increased mapping ambiguity and computational cost (Jain et al. 2021; Mokveld et al. 2020; Sirén et al. 2020). This phenomenon is paradoxical because pangenome graphs are designed to provide more available non-reference sequence paths and avoid single-reference bias. In practice, selecting a reduced variant set before simply adding all of them into the graph can help balance accuracy and computational overhead. Such variant selection can be based on criteria such as allele frequency, local density, repetitiveness, genomic location, haplotype information, and models of their integration. Algorithms like FORGe (Pritt et al. 2018), VF (Jain et al. 2021), and GraphSlimmer (Tavakoli et al. 2024) have mathematically modeled these criteria to optimize the inclusion of variants in the graph.

### High heterozygosity and polyploidy

Many horticultural crops, such as sweet orange and apple, exhibit relatively high genome heterozygosity. Unlike in human genomes, there are relatively few haplotype-resolved assemblies of heterozygous diploid plant genomes. This scarcity might explain the lack of large-scale graph pangenome studies for heterozygous diploid plants. Furthermore, many crops are allopolyploid (e.g., wheat, cotton, and rapeseed), autopolyploid (e.g., potato, alfalfa), or segmental polyploid (e.g., sweet potato). For allopolyploids, in which the constitutive subgenomes are significantly divergent or the frequency of homoeologous recombination between subgenomes is low, pangenome graph-based genotyping tools should theoretically perform efficiently. However, users of graph pangenomes should be cautious of highly similar regions between subgenomes in allopolyploids, as frequent recombination events between subgenomes can increase sequence similarity, creating more uncertainty in read mapping for variant genotypers. A recent graph pangenome study in cotton identified about 183,000 SVs in 11 assembled allotetraploid individuals; however, only 48% of these SVs were successfully genotyped using short-read data from 1158 individuals (Jin et al. 2023). Our recent work in allotetraploid rapeseed has shown a similar decrease in genotyping performance (Du et al. 2024). Therefore, specialized, subgenome-aware algorithms are required for allopolyploid genomes. For example, an autopolyploid genotyping model might improve the variant genotyping in highly similar regions occurring among subgenomes. For other complex polyploid genomes such as sweet potato (AAAABB) or sugarcane, a genotyping Hidden Markov Model integrating both diploidy and autopolyploidy can be considered for different subgenomes.

To our knowledge, there are no available tools designed for pangenome graph-based variant genotyping in autopolyploid genomes. Autopolyploids possess more than two sets of homologous chromosomes, and the number of possible genotypes at a variant locus depends on the ploidy level and the number of distinct alleles. For example, the genotypes for a biallelic locus (reference allele: A, alternative allele: a) in an autotetraploid genome can be AAAA, AAAa, AAaa, Aaaa, or aaaa. The increased number of possibilities results in higher requirements for sequencing reads and makes it more difficult to distinguish between different candidate genotypes, especially for genomes with high ploidy levels. Even worse, autopolyploid genomes often contain considerable multi-allelic loci, as revealed by the haplotype-resolved assembly of a tetraploid potato genome (Sun et al. 2022), further increasing the number of possible genotypes. To address these challenges, graph-based genotyping models can be adapted in a manner similar to the development of linear polyploid reference-based methods, such as GATK and Octopus (Poplin et al. 2018; Cooke et al. 2021). Additionally, genotyping with short reads requires comparatively higher sequencing coverage to ensure that a sufficient number of reads covers each haplotype at variant loci. Alternatively, the use of long reads can improve genotyping performance, although this approach comes with increased costs.

## Conclusions and remarks

Continuous innovations and improvements in sequencing technologies have led to a surge in genomic and pangenomic studies. By leveraging the advantages of representing non-reference sequences and benefiting downstream analyses, graph pangenomes are now widely applied across human, animal, and plant genomics. In recent years, the bioinformatics community has made significant progress in developing pangenome graph-related methods, encompassing construction, indexing, storage, visualization, read-to-graph alignments, variant calling, genotyping, and other downstream analyses. Nonetheless, challenges remain in some of these areas, especially in plant studies. As increasing numbers of crop genome assemblies become available, developers must consider how to create efficient and scalable algorithms to handle these vast datasets (Heumos et al. 2024).

Although the principles and algorithms of graph pangenomes are still evolving, they have already demonstrated their value in plant genomic studies. Examples of graph pangenomes in crops have revealed previously hidden genetic diversity and missing heritability, along with functional loci associated with important traits that have been selected for during domestication and breeding. Remarkably, some candidate loci, identified using graph pangenomes, are not detectable through traditional approaches, based on linear references and small variants, underscoring the critical role of graph pangenomes in accelerating molecular breeding. However, biologists are generally more familiar with linear reference-based analyses due to their ease of use and intuitive nature. Therefore, we conclude that graph pangenomes will not fully replace linear reference genomes but, rather, they can be prioritized in many routine genomic analyses. Looking forward, graph pangenomes are poised to drive advancements in various functional -omics fields, including plant transcriptomics, epigenomics, and 3D genomics.

## Data Availability

Data sharing is not applicable for this article, as no specific datasets were generated or analyzed during the current study.
